# A seated virtual exercise program to improve cardiovascular function in adults with chronic neurological impairments: a randomized controlled trial

**DOI:** 10.3389/fresc.2025.1477969

**Published:** 2025-03-26

**Authors:** Devina S. Kumar, Amy Bialek, Ayushi A. Divecha, Rachel M. Garn, Lydia E. J. Currie, Kathleen M. Friel

**Affiliations:** ^1^ Clinical Laboratory for Early Brain Injury Recovery, Burke Neurological Institute, White Plains, NY, United States; ^2^ Physical Therapy Department, University of Rhode Island, Kingston, RI, United States; ^3^ Department of Rehabilitation and Human Performance, Icahn School of Medicine at Mount Sinai, New York, NY, United States; ^4^ Brain and Mind Research Institute, Weill Cornell Medicine, New York, NY, United States

**Keywords:** tele-exercise, virtual, chronic neurological impairments, cardiovascular function, rehabilitation

## Abstract

**Background:**

Individuals with chronic neurological impairments often face significant barriers to regular exercise such as limited access to facilities, transportation challenges, and safety concerns. Tele-exercise has emerged as a potential solution to these challenges, particularly in the context of the COVID-19 pandemic. This study aimed to investigate the effects of a seated home-based tele-exercise regimen on cardiovascular function in adults with chronic neurological impairments.

**Methods:**

In this virtual randomized controlled trial, 63 participants with Chronic Neurological Impairments were randomized into either a synchronous group that attended live online exercise sessions via Zoom, or an asynchronous group that accessed pre-recorded exercise sessions. Both groups completed three 45 min sessions per week focused on moderate to high-intensity seated exercises over 12 weeks. Primary outcomes including Heart Rate (HR) Recovery (HRR), HR at Rest (HR Rest) and HR at peak exercise (HR Max) were averaged across three sessions at baseline, mid-study, and end of study. Secondary outcomes, including satisfaction with the equipment and virtual format were assessed using custom-designed surveys, while exercise motivation, enjoyment, and quality of life were measured using standardized instruments.

**Results:**

The analysis of HRR across the pre (*p* = 0.57), mid (*p* = 0.7), and post time points (*p* = 0.61) revealed no statistically significant differences between the synchronous and asynchronous groups. HR Rest and HR Max did not change over time. The synchronous group showed higher exercise motivation compared to the asynchronous group (*p* = 0.0001). Satisfaction with the virtual format was high, with 90% of participants reporting satisfaction with the use of the Polar heart rate monitor and 84% with Zoom.

**Conclusion:**

While no significant cardiovascular improvements were observed, the study highlights the feasibility of a virtual, seated exercise program for individuals with chronic neurological impairments. The higher reported exercise motivation in the synchronous group suggests that live, interactive sessions may be more engaging for participants. These findings underscore the potential of tele-exercise programs to provide accessible, home-based interventions, though further research is necessary to assess their long-term impact on cardiovascular health and overall well-being.

**Clinical Trial Registration:**

identifier (NCT04564495).

## 1 Introduction

Cardiovascular health is a critical concern for individuals with Chronic Neurological Impairments (CNI), as they are at an elevated risk for conditions such as coronary artery disease and increased mortality (1–3). Exercise is a cornerstone of maintaining cardiovascular health with the American College of Sports Medicine recommending at least 150 min of moderate-intensity exercise or 75 min of vigorous-intensity aerobic exercise per week (4). However, individuals with CNI often struggle to meet these guidelines due to barriers such as inadequate access to specialized exercise facilities, insufficient transportation options, and a scarcity of tailored exercise programs designed to meet their specific needs (5–7). These challenges are compounded by safety concerns of injury or unexpected cardiovascular events during exercise (8). While alternative options such as virtual fitness classes, home workout routines, and outdoor activities exist, low confidence to exercise alone can decrease participation. As a result, individuals with CNI adopt sedentary lifestyles, which not only decreased quality of life, but also contribute to secondary health conditions (7, 9, 10).

Heart Rate Recovery (HRR), a marker of cardiovascular function and autonomic nervous system balance (11). Resistance training has been associated with increased parasympathetic activity, leading to faster HRR offering a useful measure to track exercise response and adaptation (12). It can also be utilized to curate optimal exercise intensity for training (13). Despite its importance, there is a lack of evidence on how to safely and effectively improve HRR in individuals with CNI, especially using exercise regimens adapted to their unique physical abilities and environmental limitations.

Seated exercises offer a feasible and safe approach to promoting physical activity in individuals with CNI. These exercises accommodate mobility challenges while allowing for cardiovascular engagement, reducing the risk of falls or other injuries. Seated regimens are particularly relevant for individuals with limited weight-bearing capacity, spasticity, or balance impairments, providing a structured format for engaging in aerobic activity while addressing these unique needs in a controlled environment. However, there remains a need for structured research on the efficacy of seated exercise program in improving cardiovascular outcomes such as HRR for this population.

The COVID -19 pandemic exacerbated physical inactivity worldwide by limiting outdoor activities and access to gyms (14, 15). Additionally, fear of infection and social distancing guidelines deterred many from engaging in group exercise activities (16, 17). During this period, telecommunication technologies emerged as an innovative tool for delivering exercise programs remotely, allowing individuals to engage in guided physical activity from their homes (18–20). Tele-exercise programs have been shown to improve motor function, balance, and quality of life for neurological populations (15, 17, 21–23). However, little is known about their impact on cardiovascular outcomes, such as HRR, in this population (24–26).

To address these gaps, the objective of the study as outlined in our previously published protocol (27) was to assess the effectiveness of a seated tele-exercise program on improving cardiovascular function in adults with chronic neurological impairments. A key focus was the measurement of HRR, a critical parameter for assessing cardiovascular health, risk stratification, exercise prescription, intervention response, and quality of life outcomes in this population. HRR, defined as the difference between the peak heart rate achieved during exercise and the heart rate measured at intervals up to five minutes post-cool-down, serves as a marker of parasympathetic tone. Resting HR (HR Rest) before session initiation and Maximum HR (HR Max) recorded during peak exercise were also recorded to determine the effects of exercise on heart rate adaptation. The secondary objective was to explore the impact of synchronous (“live”) and asynchronous (“offline”) tele-exercise formats on participant experience, engagement, and motivation to exercise. This was assessed using validated outcome measures, including the Reason to Exercise Inventory (REI), the Physical Activity Enjoyment Scale (PACES), the Physical Wellness Scale (PWS), and the 36-Item Short Form Health Survey (SF-36). By analyzing these outcomes, the study aimed to understand how different tele-exercise delivery modes influence participant experience and engagement. The study was not designed to establish equivalence between synchronous and asynchronous formats but to identify their unique strengths and practical applications in real-world settings. The findings are intended to guide future research focused on optimizing tele-exercise programs, enhancing their accessibility, and improving their effectiveness.

## 2 Materials and methods

### 2.1 Study design

This study was a parallel randomized controlled trial to examine the effects of a 12-weeks tele-exercise program provided to a synchronous or asynchronous group of adults with CNI. This study was conducted virtually by the researchers and the Sabrina Cohen Foundation to promote health and wellness from January 2021 (first consent) to June 2021 (end of the intervention). Due to the study's inherent design, blinding of study team members and participants to group allocation was not possible. All participants signed the informed consent virtually on RedCap. The study was approved by the BRANY Institutional Review Board and registered on ClinicalTrials. Gov (NCT04564495). A comprehensive protocol has been previously published (27).

### 2.2 Participants

Individuals diagnosed with chronic neurological impairments were included in the study if they:

• Had been diagnosed for >6 months.
• 18–75 years old.
• Able to sit for at least 1 h.
• Stable Heart Rate (HR) defined as values within normal resting range of 60–100 beats per minute
• Stable BP defined as the normal resting value of 120/80 or as specified by their medical practitioner in the clearance letter
• Had medical clearance to participate in moderate intensity exercises.
• Could don and doff a wrist HR monitor with or without assistance.
• Could maintain daily exercise and physical activity during the study.
• Had no other neurological, medical, or cognitive impairments.
• Could access the internet and use the cloud-based Zoom conference platform.
• Could speak and understand English.
• Participated in structured exercises 2 days or less per week.
• Had a caregiver or supervisor during exercise for safety.

Participants were excluded if they:

• Had unstable or uncontrolled medical conditions.
• Had medical issues that prevented safe participation.
• Could not follow simple two-step commands.
• Participated in a structured exercise program exceeding 150 min per week
• Participated in any formal exercise program for more than two days per week.

### 2.3 Randomization Procedure

**Figure 1:**
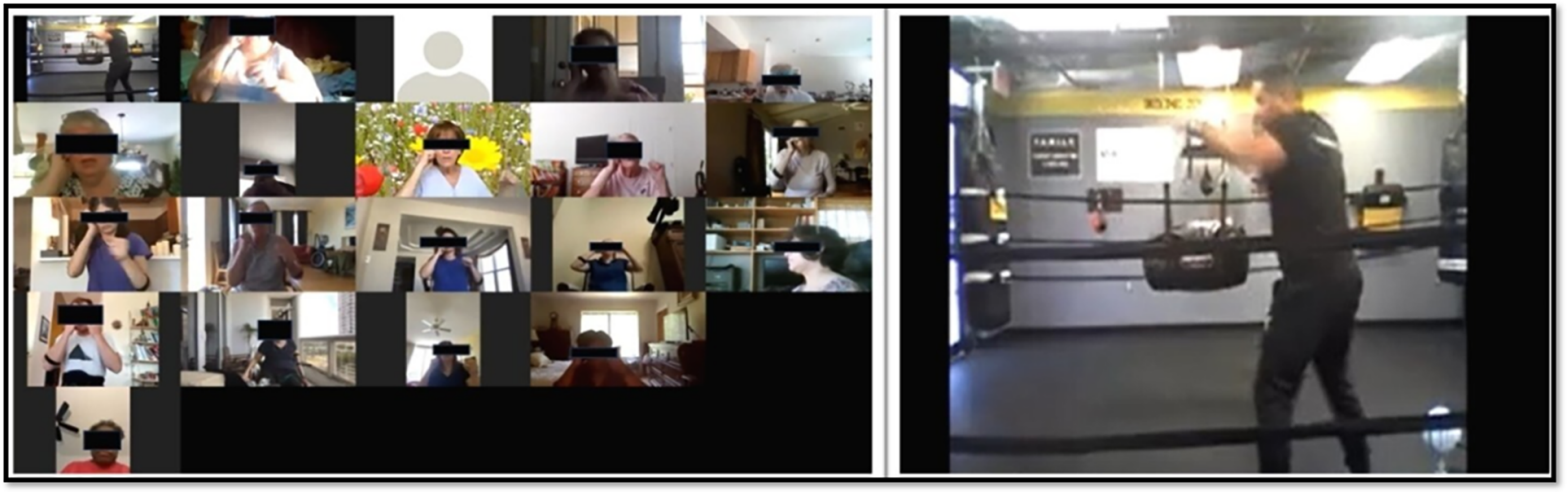
Comparison of screen views of the synchronous group (left) and asynchronous group (right) during a boxing exercise session.

1:1 Block randomization was used to allocate participants to the synchronous or asynchronous group (Figure 1). A person not affiliated with the study generated a series of permuted randomized blocks with a defined block size of 4 on an electronic spreadsheet.

## 3 Intervention

### 3.1 Approach

Eligible participants received a home exercise kit including a Polar heart rate monitor with charging equipment, a blood pressure device, TheraBands, and adjustable wrist weights. Due to the virtual format of the study, all participants were trained in the proper use of the blood pressure device and the Polar heart rate monitor via Zoom with sessions lasting approximately 30–45 min minutes. Additional training was provided for participants requiring more assistance with technology.

### 3.2 Trainer

One trainer who was certified in adaptive physical training led all the exercise sessions for the synchronous group. He has experience of working with people with disabilities. Each seated aerobic session was designed and categorized by him into High-Intensity Interval Training (HIIT), Boxing, or Power Posture.

### 3.3 Exercise type

In this virtual study, to minimize the risk of falls during moderate to high intensity exercises, a seated position was deemed practical and safe for adults with CNI. All exercise sessions were standardized to last 45 min, which included a warm-up and cool-down phase. Since there is variability in strength and endurance across individuals with CNI, the virtual nature of the program prevented individualization of exercise intensity for each participant. Exercises were directed by the instructor, who advised participants to choose wrist weights or Thera Bands that aligned with their personal comfort and capacity. Participants recorded their BP and rate of perceived exertion before and after each aerobic session.

(i) High-Intensity Interval Training (HIIT) consisted of repeated intervals of high-intensity exercises lasting 10–30 s, alternating with periods of rest or active recovery. Typically, HIIT can reach 80–100 percent of maximal heart rate. In this study, the upper limit of exercise intensity was moderated by the instructor's judgment and participant feedback on rate of perceived exertion.
(ii) Boxing consisted of movements such as jabs and hooks, interspersed with periods of active recovery featuring upper extremity shuffling and relaxed movements. These exercises were structured as moderate to high-intensity aerobic activities, defined as 60%–85% of the estimated maximum heart rate, based on the participant's age.
(iii) Power Posture designed as a HIIT style class consisted of slow, gentle repetition for education of movement with the main workout focused on targeted, fast paced movements. They were structured to enhance postural awareness through exercise, stretching and range of motion.

## 4 Allotted groups

### 4.1 Synchronous group

All participants completed 36 sessions of aerobic exercise, conducted three times per week (M-W-F), with real-time interaction and feedback from the instructor. Participants were instructed to log onto a REDCap link emailed to them prior to each session, which provided access to a pre-exercise questionnaire. Additionally, all participants were required to measure their blood pressure, log onto the Polar Beat app on their phone, and wear the Polar heart rate monitor before accessing the Zoom link for the intervention. Participants had the option to keep their video on or off during the sessions. All participants were required to turn on their video at the conclusion of the exercise session. After completing the cool-down phase, they were instructed to measure their blood pressure and fill out the questionnaires provided at the end of the session. This process allowed the research team to collect valuable feedback on any adverse effects or discomforts participants may have experienced during the session. The team used this opportunity to assess participant well-being and ensure safety throughout the study.

### 4.2 Asynchronous group

Participants in the asynchronous group completed 36 sessions of aerobic exercise, three times per week, without any feedback from the instructor. Before each session, participants were required to log into a REDCap link, emailed to them weekly, to complete a pre-exercise questionnaire. They were also instructed to measure their blood pressure, log their heart rate using the Polar Beat app on their phone, and wear the Polar heart rate monitor before starting the pre-recorded exercise videos. As the session timing was flexible, participants had the option to complete the exercises at any time during the day. At the conclusion of each session, participants were instructed to complete a post-exercise questionnaire and measure their blood pressure.

## 5 Heart rate monitor

The Polar OH1 is an optical HR sensor developed by Polar ® (Polar Electro Inc., Bethpage, NY, USA). It collaborates seamlessly with the Polar Beat app to offer users extensive capabilities for heart rate monitoring and exercise tracking. The sensor is worn on the upper or lower arm where it utilizes LED lights and photodiode sensors to continuously monitor changes in blood volume and to ensure accurate heart rate detection. This data is transmitted wirelessly to compatible smartphones or other Bluetooth-enabled devices. The Polar Beat app functions in real-time to track various types of exercises such as running, cycling, and gym workouts. Users can select their activity type to commence session tracking. The app integrates with Polar Flow, an online platform, to synchronize workout data. This integration enables detailed analysis, goal establishment, and ongoing progress monitoring. It has been validated for moderate to high intensity exercises (28).

## 6 Outcome measures

### 6.1 Primary outcome measure

All HR outcomes were collected using the Polar Beat app across all 36 exercise sessions. HR Rest was measured when the participant was in a seated position for 5 min before the exercise session began. HR Max was measured during peak exercise. HRR was measured across the first five minutes after exercise ended, in 30 s increments.

### 6.2 Secondary outcome measures

All secondary outcome measures were completed by the participants directly on REDCap and were assessed at three time points: before the first session, after session 18 (midpoint), and after the final session.

*Reason for Exercise Inventory (REI)* is a 24-item scale designed to assess individuals' reasons and motives for exercising (29). Each item is rated on a 7-point scale, ranging from 1 (not at all important) to 7 (extremely important), with higher scores indicating greater motivation to exercise.

*Short Form (SF)-36* is a measure of quality of life using eight scales-Physical Functioning, Physical Role Limitations, Bodily Pain, General Health, Vitality, Social Functioning, Emotional Role Limitations, and Mental Health (30). Results are summarized in physical function. Higher scores indicate better health status.

*Physical Wellness Scale (PWS)* is a 36-item survey designed to measure an individual's perceived wellness across six dimensions: physical, psychological, emotional, intellectual, spiritual, and social (31). Each item is scored on a scale from 1 (very strongly disagree) to 6 (very strongly agree), with higher scores indicating a better perception of wellness.

*Physical Activity Enjoyment Scale (PACES)* is an 18-item scale to measure enjoyment of physical activity. Individuals rate “how you feel at the moment about the physical activity you have been doing” using a 7-point bipolar rating scale (32). Higher scores indicate more enjoyment.

### 6.3 Safety measures

*Adverse events:* Participants were instructed to log adverse events occurring during or between sessions, including pain, dizziness, or discomfort.

*Numeric Pain Rating Scale* is a screening measure used to assess current pain severity (18). Pain intensity is scored on a scale from 0–10, with 10 representing the worst pain imaginable. The NPR has demonstrated reliability and validity in neurological populations (33).

*Borg Rating of Perceived Exertion (RPE)* is a measure of perceived exertion during physical activity (34). Exertion is rated using a scale that ranges from 6 (no exertion) to 20 (absolute maximum exertion), with higher ratings indicating greater exertion.

### 6.4 Feasibility

#### 6.4.1 Attendance

In the synchronous group, attendance was determined by the number of participants who logged on for the zoom session. In the asynchronous group, attendance was determined by the number of attendees who completed the pre- and post-exercise questionnaires and logged HR activity on the PolarBeat app.

#### 6.4.2 Attrition

Measured by the number of participants who dropped out during the course of the study.

## 7 Statistical analyses

Since HR can vary on a daily basis, HR Rest, HR Max and HRR were calculated by averaging the first three sessions at baseline (1–3), mid-study (16–18), and end of study (34–36). Participants were required to attend at least 30 out of 36 sessions to be included in the analysis. Variables considered in the analysis included baseline characteristics (age, sex, diagnosis, and time since diagnosis). We used linear regression to compare the slopes of the best-fit lines between groups. The secondary outcome measures were measured before study, at midpoint, after the intervention, and at a 1 month follow up. This survey analysis included responses from participants regardless of their HR data completeness, ensuring broader insights into participant experience beyond physiological outcomes. A two-way ANOVA was used to assess group x time differences and interactions. For this study, 27 participants were required in each arm to detect a change of 20 bpm (or greater) with 80% power and 0.05 alpha. Since we anticipated a 30% drop out rate, the aim was to recruit 35 participants per group.

## 8 Results

**Figure 2:**
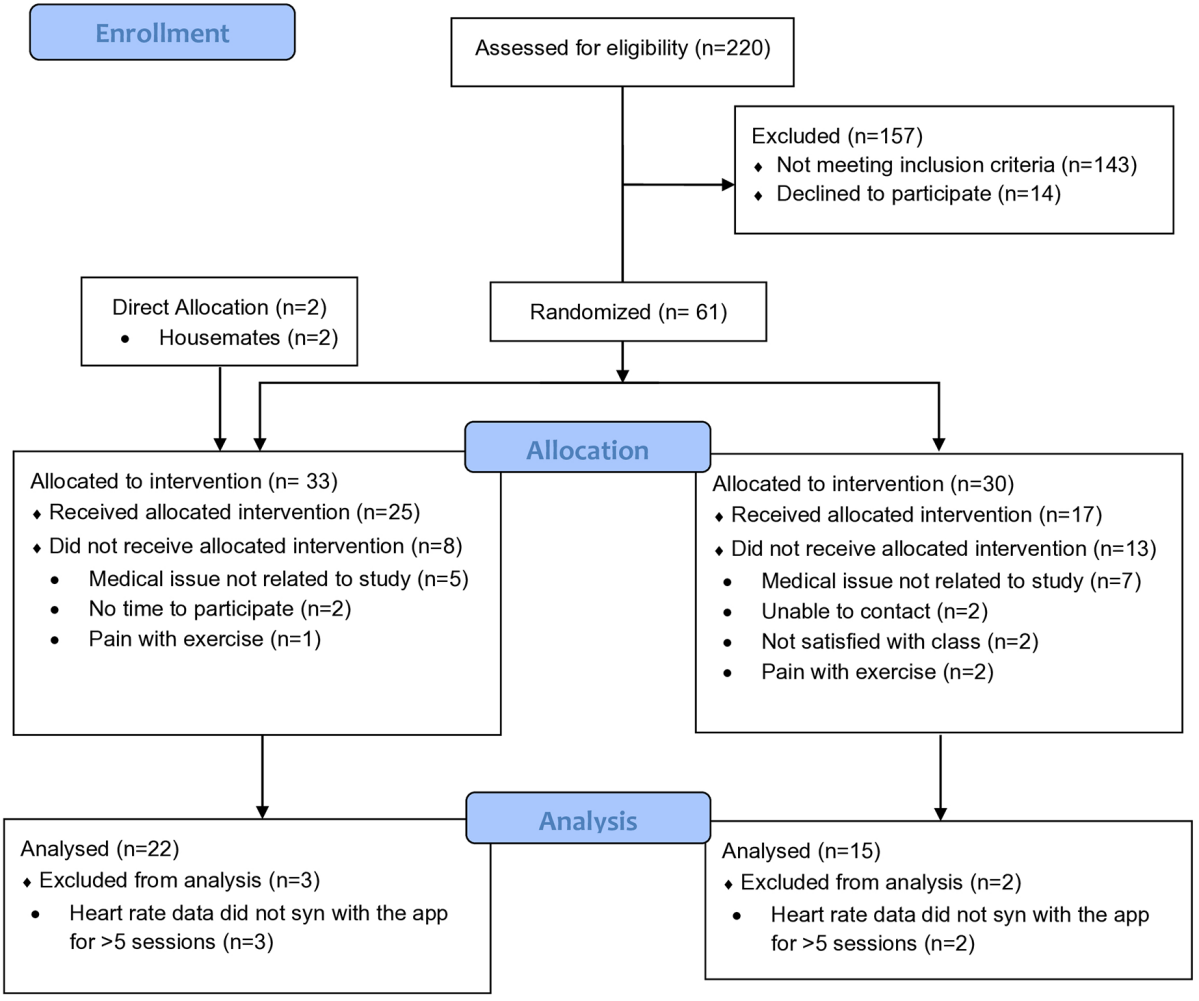
Participants flow diagram.

Of the 220 individuals that were screened, 157 were excluded from the study as they did not meet the inclusion criteria (*n* = 143) or declined to participate (*n* = 14). Of the 33 participants in the synchronous group, 25 received the allocated intervention. Eight participants did not receive the intervention because of medical issues not related to the study (*n* = 5), did not have time to participate (*n* = 2), and reported pain with exercise (*n* = 1). Of the 30 participants in the asynchronous group, 17 received the allocated intervention. Thirteen participants did not receive the intervention because of medical issues not related to the study (*n* = 7), not satisfied with the class (*n* = 2), could not be reached (*n* = 2), and reported pain with exercise (*n* = 2). HR data from three participants in the synchronous group and two participants in the asynchronous group wasn't included in the analysis as the HR activity monitor did not synchronize correctly with the Polar Beat app for more than 5 sessions. Therefore, in total, 37 participants were included in the study analysis. The participant flow diagram for the study is shown in Figure 2.

All participants were screened, of which 63 met the inclusion criteria for the study. The demographics have been summarized in Table 1. The baseline characteristics of the participants are summarized in Table 1. The average age of all participants was 55.6 ± 12 years; 75% were females, and 84.6% had a diagnosis of multiple sclerosis. Most participants were Caucasians. A pre-intervention statistical comparison between groups did not show any significant differences between groups (Table 2). The only exception was that the asynchronous group reported significantly higher PACES scores at baseline compared to the synchronous group. This suggests that there was a pre-existing difference in attitudes toward physical activity. Although the baseline disparity does not affect the primary outcomes of this study, it highlights the importance of considering individual preferences and baseline characteristics in designing tele-exercise interventions.

**Table 1:** Baseline characteristics of participants.

Demographics	Total (*n* = 37)	Synchronous (*n* = 22)	Asynchronous (*n* = 15)
Age (years; mean, SD)	55.6 (12.0)	58.9 (9.6)	51.4 (13.6)
Time since diagnosis (mean, SD)	18.3 (10.6)	15.8 (9.2)	21.6 (11.7)
Sex (*n*, %)			
Female	29 (74.4)	18 (81.8)	11 (64.7)
Male	10 (25.6)	4 (18.2)	6 (35.3)
Diagnosis (type; *n*, %)			
Multiple Sclerosis	33 (84.6)	18 (81.8)	15 (88.2)
Spinal Cord Injury/Transverse Myelitis	3 (7.7)	2 (9.1)	1 (5.9)
MTD	1 (2.6)	1 (4.5)	0
Fredrichs Ataxia	1 (2.6)	0	1 (5.9)
Neuromyelitis Optica	1 (2.6)	1 (4.5)	0
Race (type; *n*, %)			
Caucasian	34 (87.2)	20 (90.9)	14 (82.4)
African American	2 (5.1)	2 (9.1)	0
Native American	1 (2.6)	0	1 (5.9)
Asian	1 (2.6)	0	1 (5.9)
Latina	1 (2.6)	0	1 (5.9)

**Table 2:** Baseline comparison of outcome measures between groups.

Measures	Mean SD		*P* value	95% CI of the difference	
	Synchronous	Asynchronous		Lower	Upper
Primary					
HR Rest	77.32 (7.74)	74.86 (10.57)	0.44	−4.09	9.01
HR Max	112.81 (17.34)	115.04 (15.37)	0.68	−13.16	8.69
HRR	80.65 (10.17)	80.66 (11.47)	1.0	−7.46	7.47
Secondary					
REI	111.08 (20.28)	100.93 (21.53)	0.149	−3.863	24.15
PACES	61.50 (6.45)	65.53 (4.99)	0.032*	−7.7	−.366
PWS	128.5 (11.73)	124.67 (11.01)	0.303	−3.627	11.29
SF 36-PH	30.38 (28.17)	27.67 (26.91)	0.762	−15.40	20.83

HR, heart rate; HRR, heart rate recovery; REI, reason for exercise inventory; PACES, physical activity enjoyment scale; PWS, physical wellness scale; SF-36 PH, short form 36—physical function.

* Significant difference between groups *p* < 0.05

### 8.1 Feasibility

The recruitment rate for the study was 28.64%, with 63 of the 220 screened individuals enrolling. No adverse events were reported in either group. Adherence was measured by the number of sessions attended by all participants in both groups. The adherence was 67% in the synchronous group and 57% in the asynchronous group. On average, participants in the synchronous group completed 29 sessions, while those in the asynchronous group completed 26 sessions. Over the course of the study, there were 11 dropouts from the synchronous group and 13 from the asynchronous group. In a follow-up survey conducted upon study completion, 90% of participants reported being satisfied or extremely satisfied with the use of OH1, 84% with the use of Zoom, and 97% with the ease of answering surveys online.

### 8.2 Primary outcome measure

**Figure 3:**
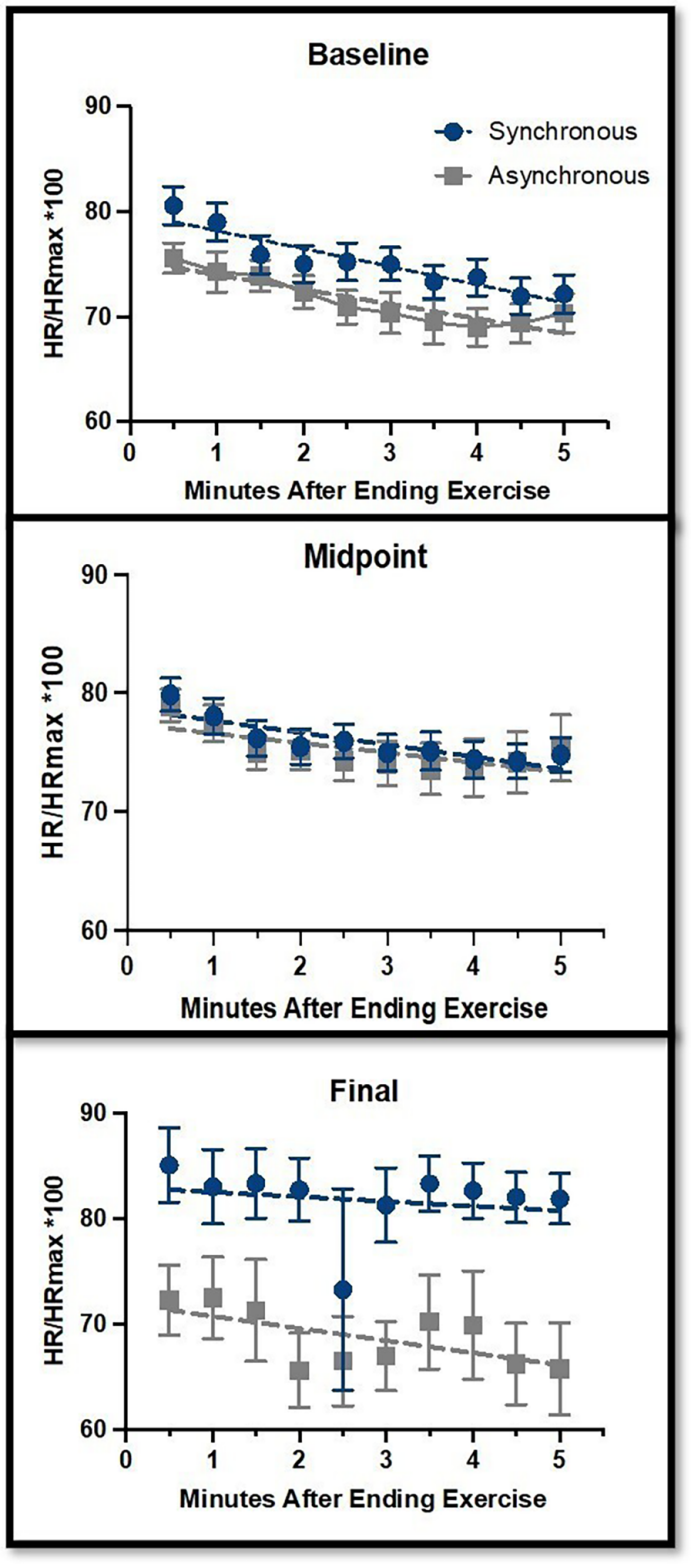
Heart rate recovery (HRR) comparisons between the synchronous (blue) and asynchronous (gray) groups at baseline, midpoint, and final sessions. The *y*-axis represents HR as a percentage of HR Max, measured from 0–5 min after completing the exercise sessions. Error bars represent the standard error of the mean. There were no significant changes across the study time points for HRR.

**Figure 4:**
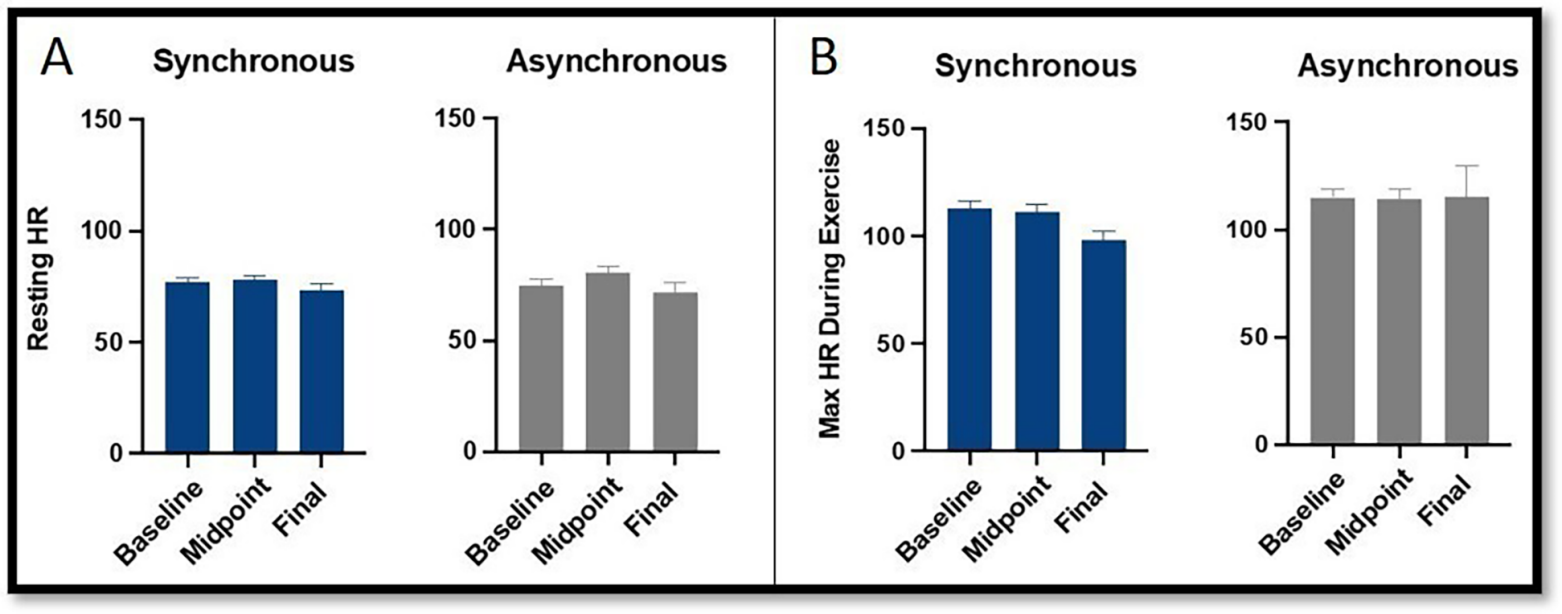
Comparison of heart rate at **(A)** rest and **(B)** peak exercise between the synchronous (blue) and asynchronous (gray) groups across baseline, midpoint, and final exercise sessions. Error bars represent the standard error of the mean. There were no significant changes across the study time points for HRR.

The results of primary outcome measures are shown in Figures 3, 4. *HRR*: For the pre [F(1,376) = 0.31, *p* = 0.57], mid [F(1,375) = 0.15, *p* = 0.70], and post [F(1,127) = 0.26, *p* = 0.61] timepoints, the slopes of the lines of each group were not statistically significantly different from one another, indicating that HRR did not differ across groups (Figure 3).

*HR Rest* (Figure 4) did not significantly change across the study timepoints [F(2,104) = 1.56, *p* = 0.22]. There was no interaction between group and timepoint [F(2,104) = 0.66, *p* = 0.52].

*HR Max* (Figure 4) did not significantly change across the study timepoints [F(2,104) = 0.18, *p* = 0.83]. There was a trend toward interaction between group and timepoint [F(2,104) = 2.74, *p* = 0.069], with the synchronous group having a slightly lower HR Max than the asynchronous group at the final timepoint (*p* = 0.064).

### 8.3 Secondary outcome measures

**Figure 5:**
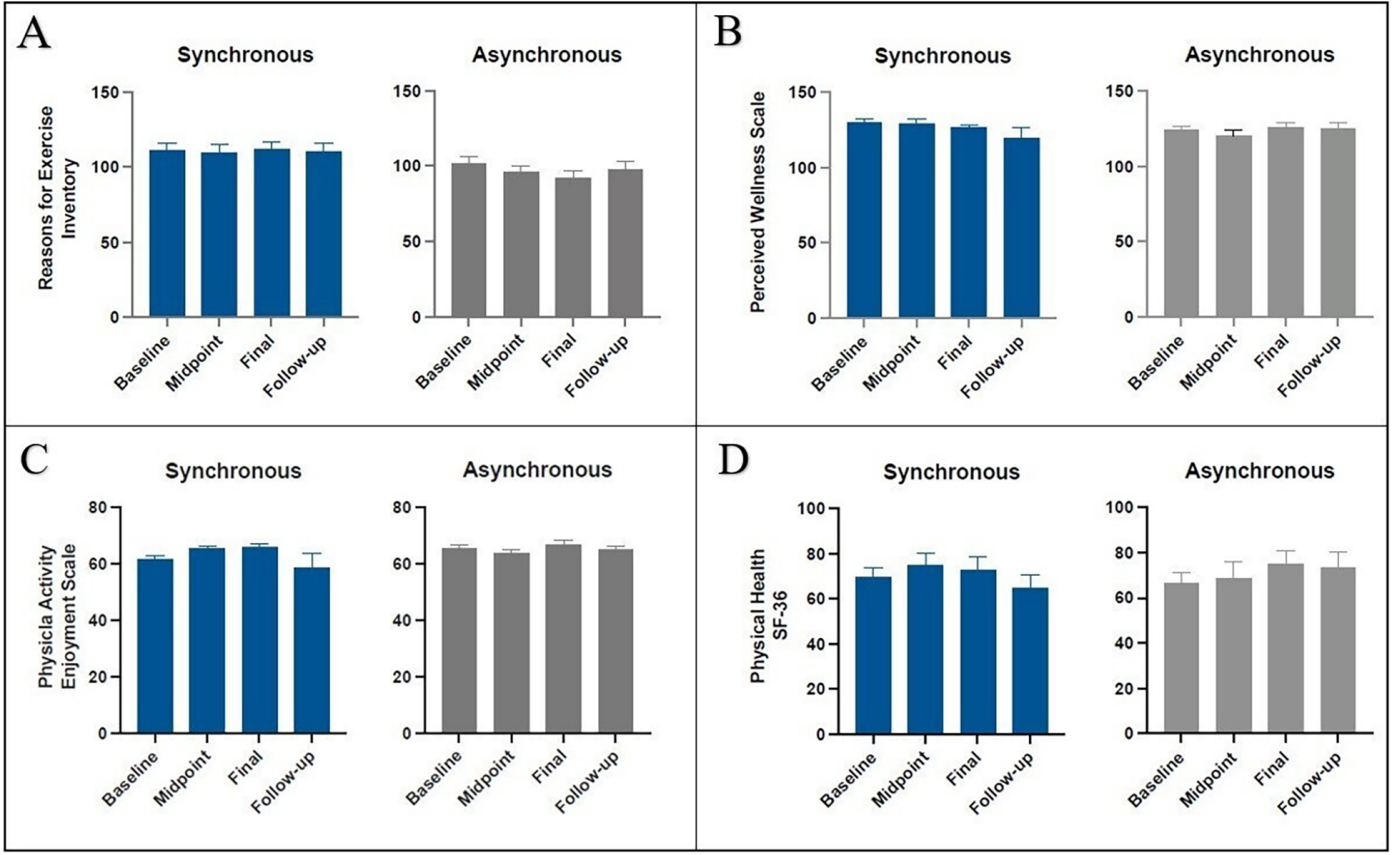
Mean change in the synchronous (blue) and asynchronous (gray) groups as measured by the reasons for exercise inventory **(A)**, perceived wellness scale **(B)**, physical activity enjoyment scale **(C)**, and physical health of SF-36 at, baseline, midpoint, and final, and follow up sessions. There was a significant change in REI (*p* = 0.0001) for the synchronous group. There were no significant changes in PWS, PACES, and SF-36 between groups and across the study time points.

The results of secondary outcome measures are shown in Figure 5. *REI* showed a significant difference between the two groups [F(1,156) = 15.15, *p* = 0.0001], with REI being higher for the synchronous group. The REI did not significantly change across the study timepoints [F(3,156) = 0.23, *p* = 0.87]. There was no interaction between group and timepoint [F(3,156) = 0.52, *p* = 0.67].

*PACES* did not significantly change across the study timepoints [F(3,156) = 0.61, *p* = 0.61]. There was no interaction between group and timepoint [F(3,156) = 1.74, *p* = 0.16].

*PWS* did not significantly change across the study timepoints [F(3,156) = 0.55, *p* = 0.65]. There was no interaction between group and timepoint [F(3,156) = 0.71, *p* = 0.55].

In the *SF-36,* the physical functioning measure did not significantly change across the study timepoints [F(3,156) = 0.51, *p* = 0.67]. There was no interaction between group and timepoint [F(3,156) = 0.67, *p* = 0.57].

## 9 Discussion

In this randomized controlled trial, we investigated the feasibility of implementing a three-month tele-exercise program conducted entirely virtually. Additionally, we evaluated the hypothesis regarding the effectiveness of seated, home-based exercise on cardiovascular function in adults with chronic neurological impairments (CNI). The study findings are discussed below.

A systematic review and meta-analysis has shown that seated exercises have a beneficial effect on cognition, strength, quality of life, and depression (35). However, their effects on cardiovascular function in adults with CNI remain unclear. This study examined the effects of moderate to high intensity upper extremity seated exercises on HRR, HR Rest and HR Max finding no significant differences between the groups at any of the measured timepoints (pre, mid, and post-intervention). This suggests that the seated home-based exercise program, regardless of whether it was a synchronous or asynchronous group, did not differentially impact any HR outcomes. A trend towards a lower HR Max in the synchronous group may be attributed to the intervention duration or the specific nature of the seated exercises, which was insufficient to drive cardiovascular adaptations. Prior research in MS has shown minimal improvements in cardiac autonomic control even after six months of exercise, highlighting the challenge of addressing autonomic dysfunction in this population (36–38). Longer interventions and multifaceted approaches may be needed to achieve cardiovascular benefits.

Since we did not assess the severity of the disease by group at baseline, we don't know if severity differed by groups enough to contribute to the outcome. Another potential reason why significant changes were not found in these measures or the secondary measures discussed below is the moderate adherence rate of both groups of participants. While the moderate adherence was disappointing, it assisted us in understanding the limitations of our virtual exercise program, discussed below.

A significant difference was observed between the synchronous and asynchronous groups, with the synchronous group reporting higher REI scores. This suggests that participants in the synchronous group perceived their exertion as higher, which could be from the real-time supervision and feedback provided during exercise sessions. However, REI did not change significantly over time, indicating consistent perceived exertion levels throughout the study. The unchanged PWS, PACES, and Sf-36 suggest that the seated home-based exercises did not significantly impact on the perceived well-being or enjoyment levels of the participants. This was an unexpected outcome as we hypothesized that individuals in the synchronous group would report greater satisfaction and enjoyment levels. Once again, the relatively short duration of this intervention may have prevented detection of a significant difference. Perhaps providing long term engagement through different exercise forms, optimization of training protocols to match specific needs, and establishing good practices early on might provide more favorable outcomes in adults with CNI (39, 40).

This study also demonstrated the feasibility of a completely virtual seated tele-exercise program. While the synchronous group had slightly better adherence, both groups maintained a reasonable level of participation. The dropout rates observed in this study (33% in the synchronous group and 43% in the asynchronous group) reflect a significant challenge in maintaining participant engagement in a fully virtual exercise program for individuals with chronic neurological impairments. While these rates are consistent with similar tele-exercise interventions, the reduced final sample size potentially limited the statistical power to detect significant differences between groups. This may explain the lack of observed differences in HRR and other secondary outcomes, despite the higher exercise motivation reported by the synchronous group (*p* = 0.0001).

In the follow-up survey, both groups reported high satisfaction rates in in the use of Zoom and Polar OH1 technology which reflects similar positive experiences that addressed barriers to tele-healthcare (41, 42). Thus, providing seated exercises through a virtual platform may be a powerful tool to combat the sedentary lifestyles of individuals with restricted mobility. While we aimed to enroll adults with a variety of disabilities, we witnessed the power of patient advocacy during our recruitment process. We connected with an MS organization that advertised our study to their membership and mentioned our study in a podcast. Soon after, we received many inquiries from people with MS. This is an important lesson for clinical researchers, to engage with patient advocacy groups.

This study had several limitations. Although 220 potential participants were screened, only 63 participants met the inclusion criteria. A larger sample size could have provided more robust data and increased the power of the statistical analyses, potentially revealing significant differences that were not detected in this study. Most of the participants were caucasians with multiple sclerosis. This limits the generalization of the results to other races and chronic neurological conditions. Feasibility studies with larger and more diverse populations could help improve the robustness and strength of the findings. The adherence rate was moderate for both groups. Future studies should aim to address this challenge through tailored strategies to increase adherence and decrease attrition. These could include integrating more personalized support, such as regular check-ins with the asynchronous group. Additionally, the seated home-based exercises might not have been intense enough to elicit significant cardiovascular improvements. Future studies must consider incorporating exercise intensities of varying levels to determine their effect on cardiovascular health. Although this study primarily focused on cardiovascular outcomes and psychosocial variables, additional physical measures, such as grip strength, may provide a more comprehensive evaluation of physical function. Since neurological pathologies are so complex in terms of clinical manifestations, it is necessary that therapeutic exercise be supervised and implemented by a physical therapist as a health professional. Lastly, this study focused on comparing the outcomes of synchronous vs. asynchronous groups. The absence of a no-intervention control group limits the ability to draw conclusions about the efficacy of the tele-exercise program relative to no exercise, Future research should include a control group to establish the baseline effects of such interventions and to further validate their impact on cardiovascular and engagement outcomes.

## 10 Conclusion

This study demonstrated that a virtual, seated tele-exercise program is feasible for individuals with chronic neurological impairments, with greater motivation observed in the synchronous group. However, no significant differences were found in cardiovascular outcomes or other physical and psychosocial measures between the synchronous and asynchronous groups. These results highlight the potential of synchronous tele-exercise to improve engagement but underscore the need for further studies with larger sample sizes and control groups to validate its effects on cardiovascular health and social outcomes.

## Data Availability

The raw data supporting the conclusions of this article will be made available by the authors, without undue reservation.
